# USP18 directly regulates Snail1 protein through ubiquitination pathway in colorectal cancer

**DOI:** 10.1186/s12935-020-01442-1

**Published:** 2020-07-28

**Authors:** Fakun Huang, Chengying Zheng, Longkai Huang, Changqing Lin, Jiaxing Wang

**Affiliations:** Department of Gastrointestinal Surgery, The First Affiliated Hospital of Fujian Medical University, 20 Chazhong Road, Fuzhou, 350000 Fujian People’s Republic of China

**Keywords:** Colorectal cancer, EMT, Snail1, USP18, qRT-PCR

## Abstract

**Background:**

Colorectal cancer (CRC) is one of the most common digestive malignant tumors in the world. Ubiquitin-specific peptidase 18 (USP18) plays a regulatory role in tumorigenesis, and abnormal expression of Snail1 is also believed to be related to tumorigenesis. However, whether USP18 could affect colorectal cancer through Snail1 remains unclear. This study was designed to investigate the role of USP18 in colorectal cancer.

**Methods:**

USP18 protein and mRNA abundance in clinical tissues and five cell lines were analyzed with quantitative real-time PCR (qRT-PCR) and western blot. USP18 overexpression-treated DLD1 cells and USP18 knockdown-treated SW480 cells were used to study cell proliferation, migration, invasion, and the expression of epithelial-mesenchymal transformation (EMT) biomarkers. Moreover, ubiquitination-related Snail1 degradation was detected with qRT-PCR and western blot. The relationships between USP18 and Snail1 were investigated with western blot, co-immunoprecipitation, migration, and invasion.

**Results:**

USP18 was highly expressed in colorectal cancer tissues. Overexpression of USP18 could promote proliferation, colony formation, migration, and invasion of colorectal cancer cells. Overexpression of USP18 effectively promoted cell survival after treatment with three different chemotherapy drugs. Moreover, USP18 could regulate Snail1 degradation through ubiquitination pathway. Furthermore, we demonstrated that Snail1 could effectively reverse the influence of USP18 on cell proliferation, migration, invasion, and EMT of CRC cells.

**Conclusion:**

USP18 could promote the proliferation, migration, and invasion of colorectal cancer by deubiquitinating and stabilizing the Snail1 protein in colorectal cancer.

## Background

As one of the most common malignant tumors, colorectal cancer (CRC) remains the third most incident cancer, with the incidence of colon cancer rising rapidly worldwide [1]. Previous data showed that among colorectal cancer patients, 25% were often accompanied by distant metastases, and about 40–50% who had not been found with primary colorectal cancer metastasis eventually developed distant metastasis. Moreover, the median survival of untreated colorectal cancer metastases in advanced patients was only 5–6 months [2, 3]. The occurrence and development of colorectal cancer is a process of multi-gene participation and multi-stage evolution. A current research indicates that colorectal cancer is a sequence evolutionary process in which adenomatous polyps eventually become cancerous and metastasize to the primary cancer nest [4, 5]. The inactivation of tumor suppressor genes (e.g., APC, TP53 and TGFBR2), and the mutation of oncogenes (e.g., RAS, BRAF and PI3KCA), which can inhibit or activate downstream-related signaling pathways and ultimately lead to the occurrence of adenomatous polyps and malignant tumors, are involved in the process [6]. Surgical treatment, chemotherapy, radiotherapy and other common clinical and technical methods are difficult to effectively treat late metastatic tumors, which eventually cause 90% cancer patients death [7]. Therefore, finding new biomarkers and further understanding the molecular mechanism may help prevent and treat colorectal cancer.

Epithelial-mesenchymal transformation (EMT) refers to the transformation of epithelial cells into cells with mesenchymal characteristics [8]. The process includes the loss of epithelial markers such as E-cadherin and cytokeratin, accompanied by increased expression of interstitial markers such as N-cadherin, vimentin and fibronectin [9]. Previous studies suggested that EMT could cause a variety of changes in cells, such as enhanced ability of cell migration and invasion, and enhanced resistance to apoptosis and senescence, which play a very important role in the formation of tumor metastases [10].

Snail1 is considered a key factor in the aggressive expression of tumors for its critical role in the EMT pathway associated with tumor metastasis [11]. Studies showed that Snail1 expression was significantly higher in non-small cell lung cancer tissues than in normal non-cancer tissues, which suggested a possible reaction of Snail1 in tumor [12]. Moreover, in normal tissues Snail1 gene was silent, but in tumor tissues its expression was up-regulated, and exerted a functional role by controlling the expression of related proteins [13]. Therefore, it’s still worth exploring the detailed molecular mechanism of Snaill in CRC.

Specification peptidase 18 (USP18) is a depolymerase of the ubiquitin-like modified enzyme system that can reduce the modification effect of ISG15 on the target protein by removing ISG15 from the bound target protein, which is called “deubiquitination” and has a regulatory effect on the body’s multiple signaling pathways and homeostatic maintainance [14, 15]. Recent studies showed that USP18 could also affect tumorigenesis by regulating interferon production and immune cell function, and recent researches found its expression in various tumors [16, 17]. The deletion of the USP18 gene induced the expression of exogenous apoptosis-related genes such as TRAIL by activating the I-IFN-related signaling pathway [18]. The knockdown also inhibited the EGFR expression by up-regulating miR-7, which further inhibited the growth of tumor cells and increased apoptosis [19]. Moreover, the lack of USP18 gene could inhibit the formation of leukemia induced by BCR-ABL virus through up-regulating the I-IFN signaling pathway [20]. However, the roles of USP18 and Snaill in CRC are still poorly studied. In this study, we explored whether USP18 affects CRC cells through regulating Snail1 ubiquitination.

## Methods

### Tissue samples and cells

Sixty colorectal cancer samples and their paired normal tissues were collected in the department of pathology of the First Affiliated Hospital of Fujian Medical University between Jan 2019 and Dec 2019. The ethics committee of The First Affiliated Hospital of Fujian Medical University had reviewed and approved all experimental protocols. All patients had read and signed the informed consent. The detached tissues were quickly frozen with fluid nitrogen and stored at − 80 °C. FHC, HCT116, SW480, DLD1, and LOVO cells were purchased from ATCC (Virginia, USA). Cells were cultured with RPMI 1640 with 10% FBS (Invitrogen, Carlsbad, CA) in a humidified chamber at 5% CO_2_, at 37 °C. SW480 cells were plated on six-well plates (5 × 10^5^ cells per well). OPTI-MEM serum-free medium (M5650, Sigma Aldrich) and Lipofectamine 2000 reagent (Thermo Fisher Scientific, USA) were used in transfection tests. The final concentration of 100 nM siRNA was introduced in this study. Meanwhile, pEZ-Lv201 Vector (Biovector, China) was employed to construct the USP18 overexpression system in the DLD1 cells. Lentiviral particles generated with a standardized protocol were used to produce the highly purified plasmids. Endo Fectin-Lenti™ and Titer Boost™ reagents (CWBio, China) were used to co-transfect DLD1 cells. The supernatant was collected after 48 h transfection and stored at − 80 °C.

### Effect of USP18 on chemotherapy sensitivity of CRC cells

Three common chemotherapy drugs (fluorouracial, doxorubicin, and cisplatin) were used. Overexpression or knockdown of USP18 in CRC cells were established as described above. Then, CRC cells were treated with different concentrations of fluorouracial (0, 20, 40, 60, and 80 g/mL), doxorubicin (0, 0.5, 2.5, 5, and 10 µM), or cisplatin (0, 10, 20, 30, and 40 µM) for 24 h. Then, the cell survival was measured using CCK-8 assay.

### qRT-PCR analysis

Total RNA was extracted with M5 SuperPure Total RNA Extraction Reagent (SuperTRIgent) (mei5bio, China). The mRNA expression was examined with the Q225 system (Kubotechnology, China). The PCR reaction contained 10μL GoldStar Probe Mixture (CWBio, China), 1μL sense primer (10 nM), 1μL anti-sense primer (10 nM), 2μL cDNA template (10 ng), and 6μL H_2_O. The program for qRT-PCR was set as follows: 95 °C, 30 s, 40 cycles (95 °C, 5 s, and 60 °C, 10 s). 2^−ΔΔCt^ cycle method was used to calculate the relative expression level of mRNAs. GAPDH was employed as the internal control. Primer sequences used were listed in Additional file 1: Table S1.

### Western blot analysis

Cellular protein in different groups was extracted with 1% PMSF a RIPA Lysis and Extraction Buffer (Beyotime, China). Sodium dodecy lsulfate–polyacrylamide gel electrophoresis was used to perform further examination. In this step, the proteins were transfered onto a polyvinylidene difluoride layer (Novus, USA). After blocking for 1 h at room temperature, the layer was brooded with anti-Rabbit USP18 (1:1000) (#4813, CST, USA), E-cadherin (1:1000) (# 3195S, CST, USA), Vimentin (1:1000) (# 5741S, CST, USA), N-cadherin (1:1000) (#13116S, CST, USA), CD133 (1:1000) (#64326, CST, USA), CD44(1:1000) (# 37259S, CST, USA), Snail1 (1:1000) (#3879, CST, USA), and GAPDH (1:1000) (#2118, CST, USA), overnight. Proteins were hatched with the corresponding secondary antibodies for 1 h at room temperature after being treated with ECL Chemiluminescence Detection Kit (PromoCell, German). The bands were observed with Chemiluminescence Imaging (Clinx Ltd., China).

### Immunohistochemical staining analysis

The immunohistochemical SP method was used to stain cancer tissue sections. Tissue sections were baked in a 60 °C incubator for 1 h, and then were subjected to multiple treatments, including immersion in xylene to dewax, gradient alcohol hydration, microwave antigen repair, and 3% hydrogen peroxide treatment. After blocking using goat serum, the sections were added in an anti-rabbit USP18 monoclonal antibody Snail1 (1:1000) (#3879, CST, USA) and incubated at 4 °C overnight. An optical microscope was used for observation.

### Migration and invasion assay

EZCell™ Cell Migration/Chemotaxis Assay Kit (24-well) (K911-12, Biovision, USA) and EZCell™ Cell Invasion Assay (Basement Membrane) (96-well Kit) (K912-100, Biovision, USA) were used to perform cell migration and invasion, respectively.

### CCK8

The differently-treated cells were digested, centrifuged and resuspended. The cells were diluted with complete medium. The cells were counted using a cell glass counting plate, and then diluted to 2000 cells/ml. 100 μL cell suspension (2000 cells/mL) was added to each well in a 96-well plate. There were 5 replicate wells in each group and the five replicates were set and observed at five-time points. Subsequently, we incubated the cells in a 5% CO_2_, 37 °C incubator overnight. Next day, 10 μL CCK-8 solution (Beyotime, China) was added to the medium. Then, the plate was incubated in a 37 °C incubator for 2 h. The absorbance at OD450 was measured.

### Immunofluorescence analysis

Cancer cells in the logarithmic growth phase were inoculated into 24-well plates with cell slides and cultured for 48 h. We discarded the medium, removed the cell slides, and washed 3 times with PBS. Sections were fixed using 4% paraformaldehyde at 4° C for 30 min. After washing 3 times with PBS (5 min/time), 0.1% Triton was used to treat sections for 10 min. Then, PBS was used to wash sections for 5 min, and goat serum was used for blocking. After washing 3 times with PBS (5 min/time), first antibody was used to cultivate sections at 4 °C overnight. Subsequently, secondary antibody was used to culture sections for 1 h at room temperature in a wet box. After washing 3 times with PBS (10 min/time), an inverted fluorescence microscope was used to observe results.

### Co-IP detection

Cancer cells in the logarithmic growth phase was used in this step. Total protein was extracted using the RIPA Lysis and Extraction Buffer (89900, ThermolFisher Scientific, USA). The beads were washed with 100 μL ice-cold buffer. 100 μL antibody binding buffer was added to spin the antibody and magnetic beads for 30 min. The beads were washed 3 times with 200 μL buffer. Cell lysate and antibody-conjugated magnetic beads were used to incubate for 1 h at room temperature and then washed 3 times with 200 μL buffer. 20 μL elution buffer was used to wash the beads once and the supernatant was collected.

### Scratch test

Differently Snail1 knocked-down cells were resuspended and counted. The scratch test insert after alcohol disinfection was carefully placed in a 12-well plate (3 replicates per group). The complete medium was used to dilute the cells to 500 cells/μL. 70 μL cell suspension was added to each well. Twenty-four hours later, the cells were gently washed twice with PBS and then, 1 ml 1% FBS medium was added. Cell status was observed under the microscope at 0 h and 24 h.

### Statistical methods

SPSS16.0 statistical software was used and data were expressed as χ ± s. Two groups were compared using the *t* test. One-way analysis of variance was used for comparison between groups. P < 0.05 was considered to be significant difference.

## Results

### USP18 gene was highly expressed in CRC tissue

**Fig. 1:**
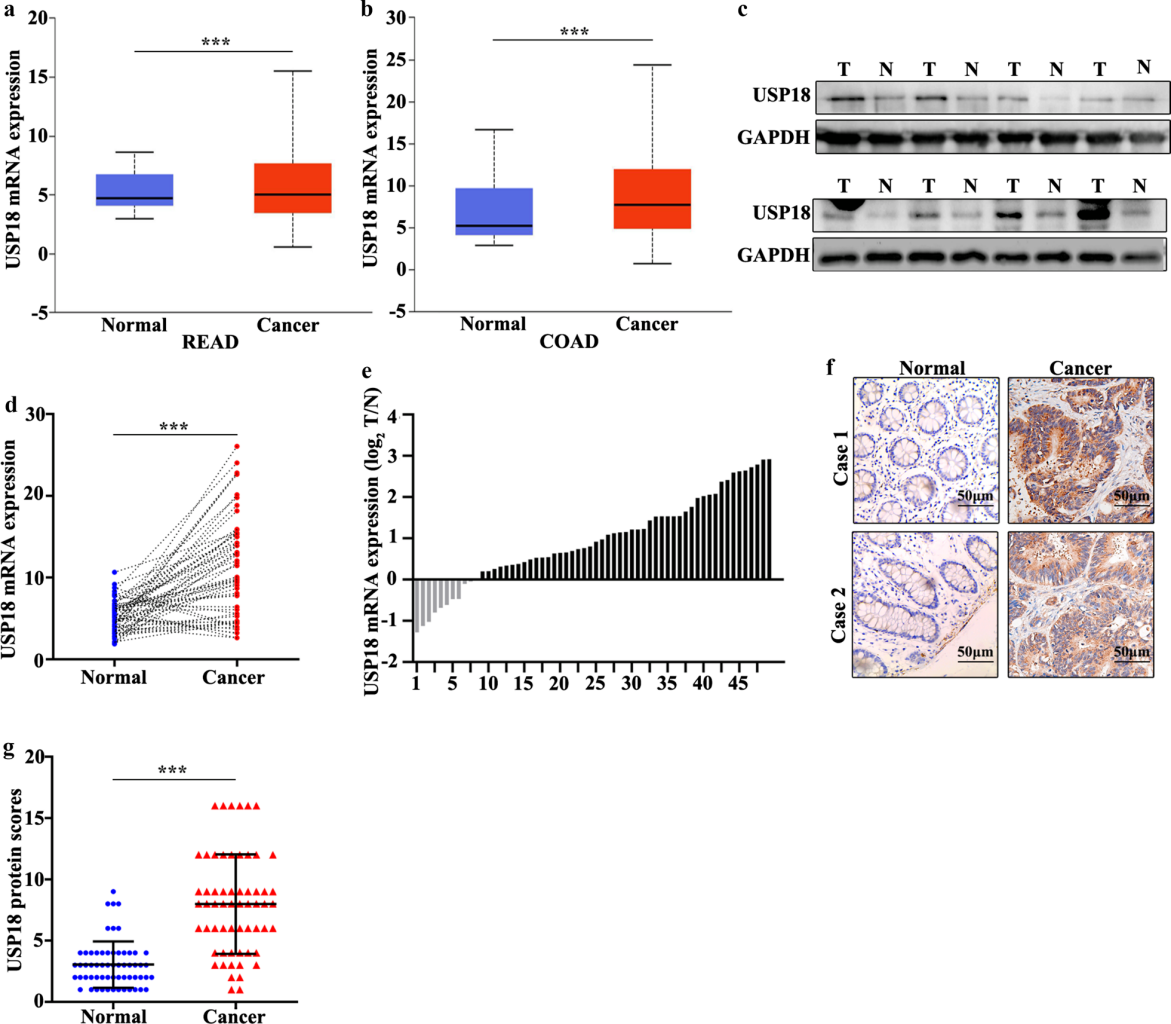
Detection of USP18 expression in colorectal cancer. **a**, **b** The expression level of USP18 was verified in UALCAN database (http://ualcan.path.uab.edu/). **c** Western blot analysis of the USP18 expression level in colorectal cancer tissues and normal tissues. **d** qRT-PCR analysis of USP18 expression level in colorectal cancer tissues and normal tissues. **e** The sample distribution analysis of the high USP18 expression in tumor tissues and adjacent tissues among 60 pairs of specimens. **f** Detection of USP18 expression levels in colorectal cancer tissues and normal tissues with IHC. **g** HC score statistics of the USP18 expression levels in 60 colorectal cancer tissues and normal tissues. ***P < 0.001

Sixty CRC patients were included in this study. The clinical features of the 60 patients were shown in the Table 1. The results suggested that significant differences could be calculated in T Stages (I–II) (P = 0.035), Metastasis (N0) (P = 0.003), and Metastasis (M0) (P = 0.025). In order to examine the expression of USP1, we first performed the detection in colorectal cancer tissues and the paired normal tissues through online dataset, western blot, qRT-PCR, and immunohistochemical staining analysis. For online dataset analysis, UALCAN database (http://ualcan.path.uab.edu/) was applied [21]. The result found that USP18 expression was higher in colorectal cancer tissues than in the paired normal tissues (P < 0.05) (Fig. 1a, b). Meanwhile, western blot analysis revealed that USP18 protein expression was significantly higher in colorectal cancer tissues than in normal tissues (Fig. 1c). qRT-PCR analysis indicated that USP18 expression was significantly higher in colorectal cancer tissues than in the paired normal tissues (P < 0.001) (Fig. 1d). Moreover, we analyzed the distribution of the high USP18 expression in colorectal cancer tissues and the paired adjacent tissues. Figure 1e suggested that 80% (40 of 50) of high USP18 expression could be detected in colorectal cancer tissues. Furthermore, immunohistochemical staining analysis indicated that USP18 expression was significantly higher in colorectal cancer tissue than in the paired normal tissues (P < 0.001) (Fig. 1f, g). In summary, USP18 expression in colorectal cancer tissues was higher than that in the paired normal tissues.

**Table 1:** Clinical features of the patients included in this study

Features	Total (n)	USP18			
		Positive	Negative	X^2^	P-value
Gender					
Male	35	29	6	0.513	0.513
Female	25	19	6		
Age (years)					
≥ 60	38	33	5	3.032	0.082
< 60	22	15	7		
T Stages					
I–II	24	16	8	4.444	0.035*
III–IV	36	32	4		
Metastasis					
N Stages					
N0	15	8	7	8.889	0.003*
N1–2	45	40	5		
M Stages					
M0	45	39	6	5.000	0.025*
M1	15	9	6		
Location					
Colon	33	25	8	0.825	0.364
Rectal	27	23	4		
Histological differentiation					
Well-moderate	34	27	7	0.017	0.896
Poorly	26	21	5

### USP18 promoted proliferation of colorectal cancer cells in vitro

**Fig. 2:**
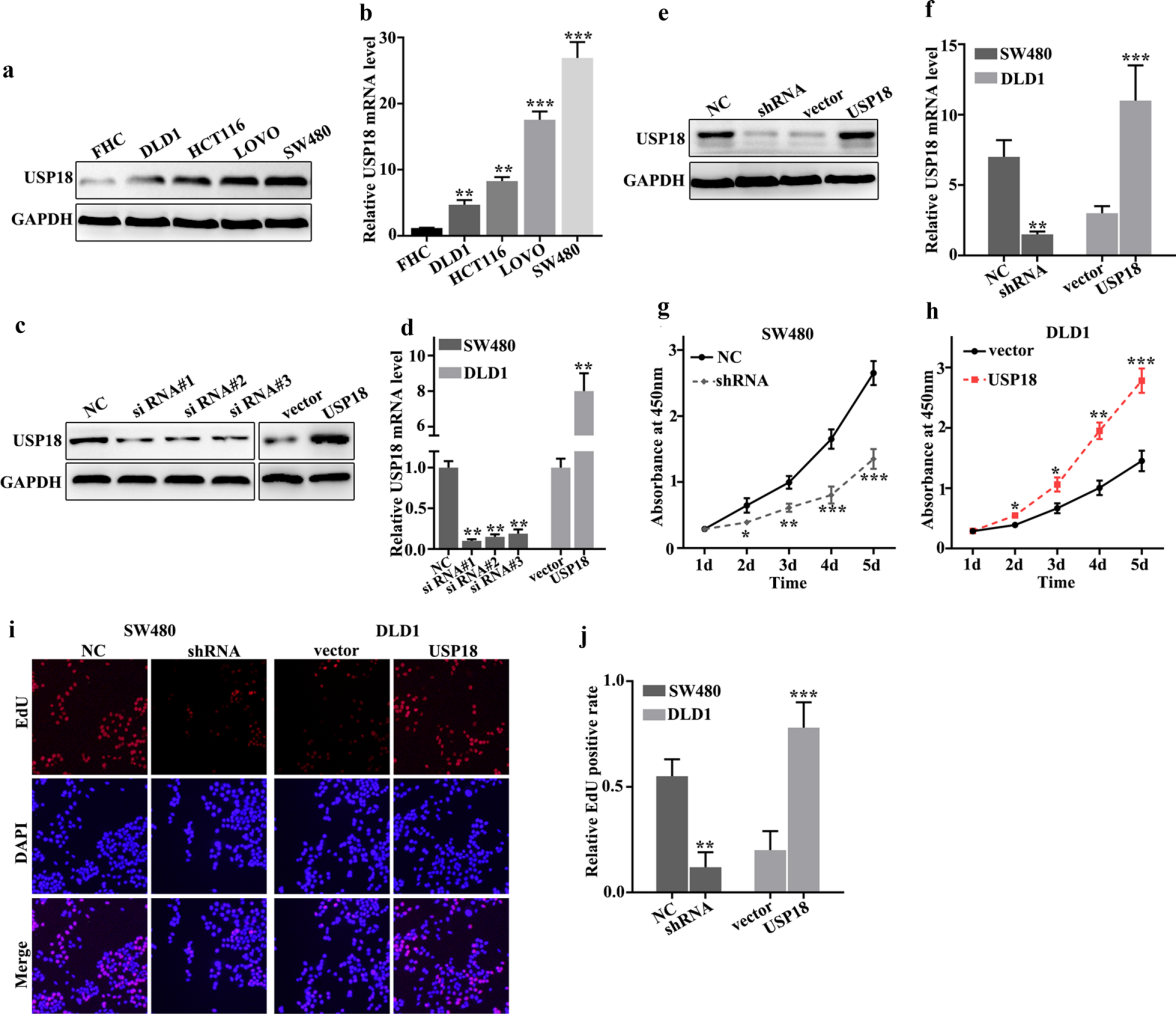
USP18 promoted colorectal cancer cell proliferation in vitro. **a**, **b** Western blot and qRT-PCR analysis of the USP18 protein and mRNA levels in normal colon epithelial cell lines and four common colorectal cancer cells. **c**, **d** Western blot and qRT-PCR were used to select sequences with better knockdown efficiency. **e**, **f** Western blot and qRT-PCR were used to identify the effects of USP18 knockdown treatment in SW480 cells and USP18 overexpression treatment in DLD1 cells. **g**, **j** CCK8 and edu experiment was employed to study the cell proliferation of USP18 knocked-down SW480 cells and USP18 over-expressed DLD1 cells. *P < 0.05, **P < 0.01, ***P < 0.001

To further probe the biological function of USP18, we studied USP18 expression in five selected cell lines, FHC, HCT116, SW480, DLD1, and LOVO. Western blot and qRT-PCR analysis of USP18 expression in five cell lines indicated that USP18 protein and mRNA expression were significantly different between each other (Fig. 2a, b). It was notable that USP18 protein and mRNA expression were lower in DLD1 cells than in other cell lines (P < 0.01), and were higher in SW480 cells than in other cell lines (P < 0.001). Therefore, DLD1 and SW480 cells were selected for further study. They were used to construct overexpression and knockdown models of USP18. Figure 2c, d showed that overexpression and knockdown of USP18 in DLD1 and SW480 cells were successfully established. siRNA #3 and USP18 vector were employed for further study. Meanwhile, we have identified the therapeutic efficiency of overexpression and knockdown in USP18 knockdown-treated SW480 cells, and USP18 overexpression-treated DLD1 cells. Figure 2e, f revealed that overexpression and knockdown system used in this study were both effective. For cell proliferation analysis, USP18 knockdown in SW480 cells could significantly reduce cell proliferation compared to normal SW480 cells on days 2, 3, 4, 5 (Fig. 2g) (P < 0.01). However, USP18 overexpression in DLD1 cells could significantly promote cell proliferation compared to vector-treated DLD1 cells on days 2, 3, 4, 5 (Fig. 2h) (P < 0.05). Moreover, we further employed edu and CCK-8 experiments to probe the change of cell number in USP18 knocked-down SW480 cells and USP18 over-expressed DLD1 cells. Figure 2i, j suggested that similar results of CCK-8 could be observed in edu experiment. USP18 knockdown in SW480 cells could significantly reduce cell proliferation while USP18 overexpression in DLD1 cells could significantly promote cell proliferation in vitro.

### USP18 regulated CRC cell migration and invasion

**Fig. 3:**
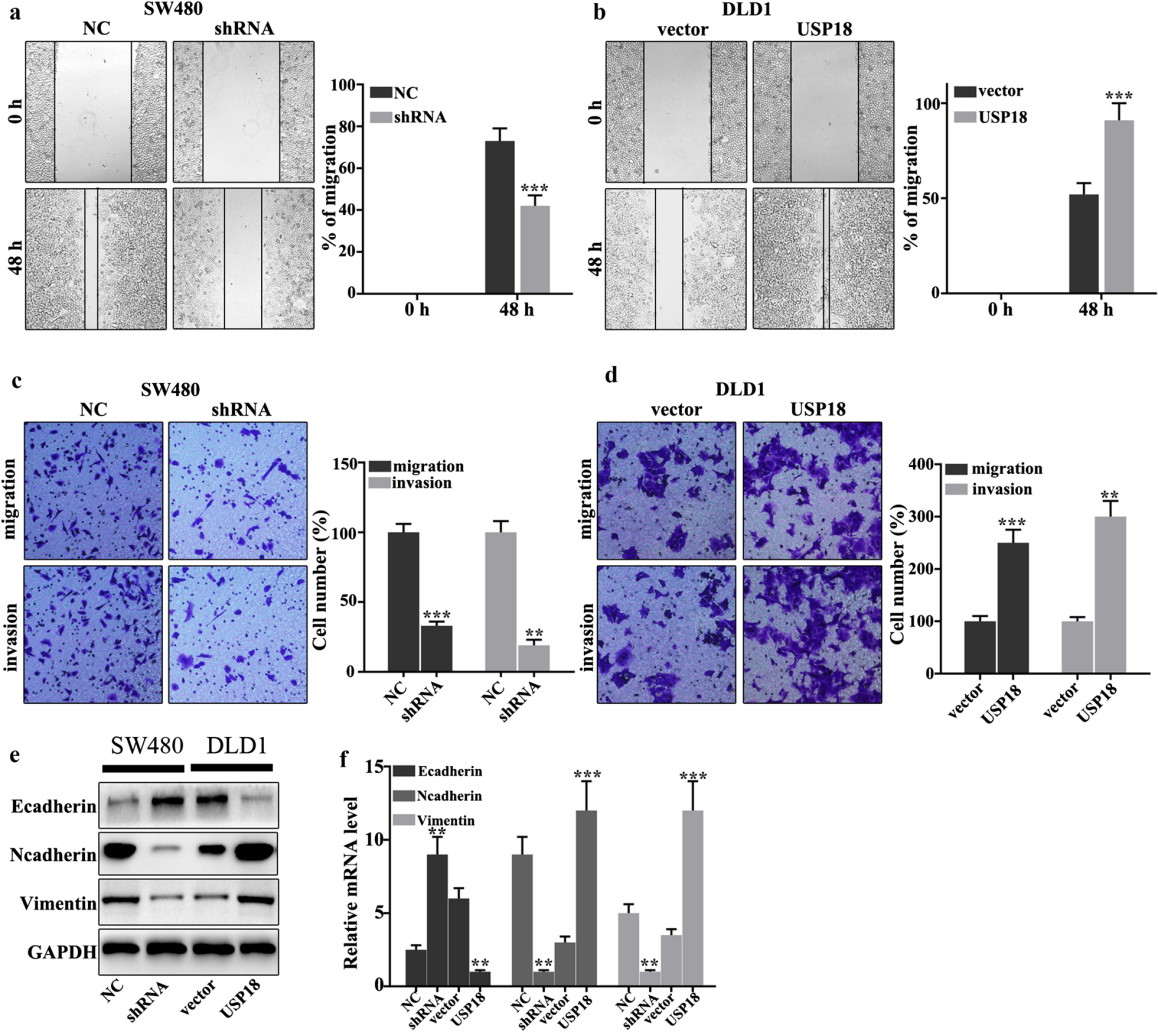
USP18 regulated the migration and invasion of colorectal cancer cells. **a**–**d** Cell scratch test and invasion assays analysis of USP18 knocked-down SW480 cells and USP18 over-expressed DLD1 cells. **e**–**f** Western blot and qRT-PCR analysis of the EMT-related proteins in USP18 knocked-down SW480 cells and USP18 over-expressed DLD1 cells. **P < 0.01, ***P < 0.001

In this work, we investigated whether USP18 could regulate colorectal cancer cells migration and invasion in USP18 knockdown-treated SW480 cells and USP18 overexpression-treated DLD1 cells. Figure 3a showed that USP18 knockdown in SW480 cells could significantly inhibit cell scratch ability compared to normal SW480 cells (P < 0.001). Meanwhile, USP18 overexpression in DLD1 cells could significantly promote cell scratch ability compared to normal DLD1 cells (P < 0.01) (Fig. 3b). Moreover, we further examined the cell migration and invasion in USP18 knocked-down SW480 cells and USP18 overeexpressed DLD1 cells. USP18 knockdown in SW480 cells could significantly inhibit cell migration and invasion compared with that in the normal SW480 cells (P < 0.01) (Fig. 3c). However, USP18 overexpression in DLD1 cells could promote cell migration and invasion compared with that in the normal DLD1 cells (P < 0.01) (Fig. 3d). Moreover, we further analyzed the protein expression of E-cadherin, N-cadherin and Vimentin. USP18 knockdown in SW480 cells could effectively inhibit the protein expression of N-cadherin and Vimentin (P < 0.01) (Fig. 3e, f), but promote the E-cadherin protein expression (P < 0.01). USP18 overexpression in DLD1 cells could effectively increase the protein expressions of N-cadherin and Vimentin (P < 0.01), but inhibit E-cadherin protein expression (P < 0.01). Therefore, USP18 overexpression in DLD1 cells could promote the protein expression of N-cadherin and Vimentin, but inhibit the protein expression of E-cadherin. Meanwhile, USP18 knockdown in SW480 cells could inhibit the protein expression of N-cadherin and Vimentin, but promote the protein expression of E-cadherin.

### USP18 affected chemotherapy sensitivity of CRC cells

**Fig. 4:**
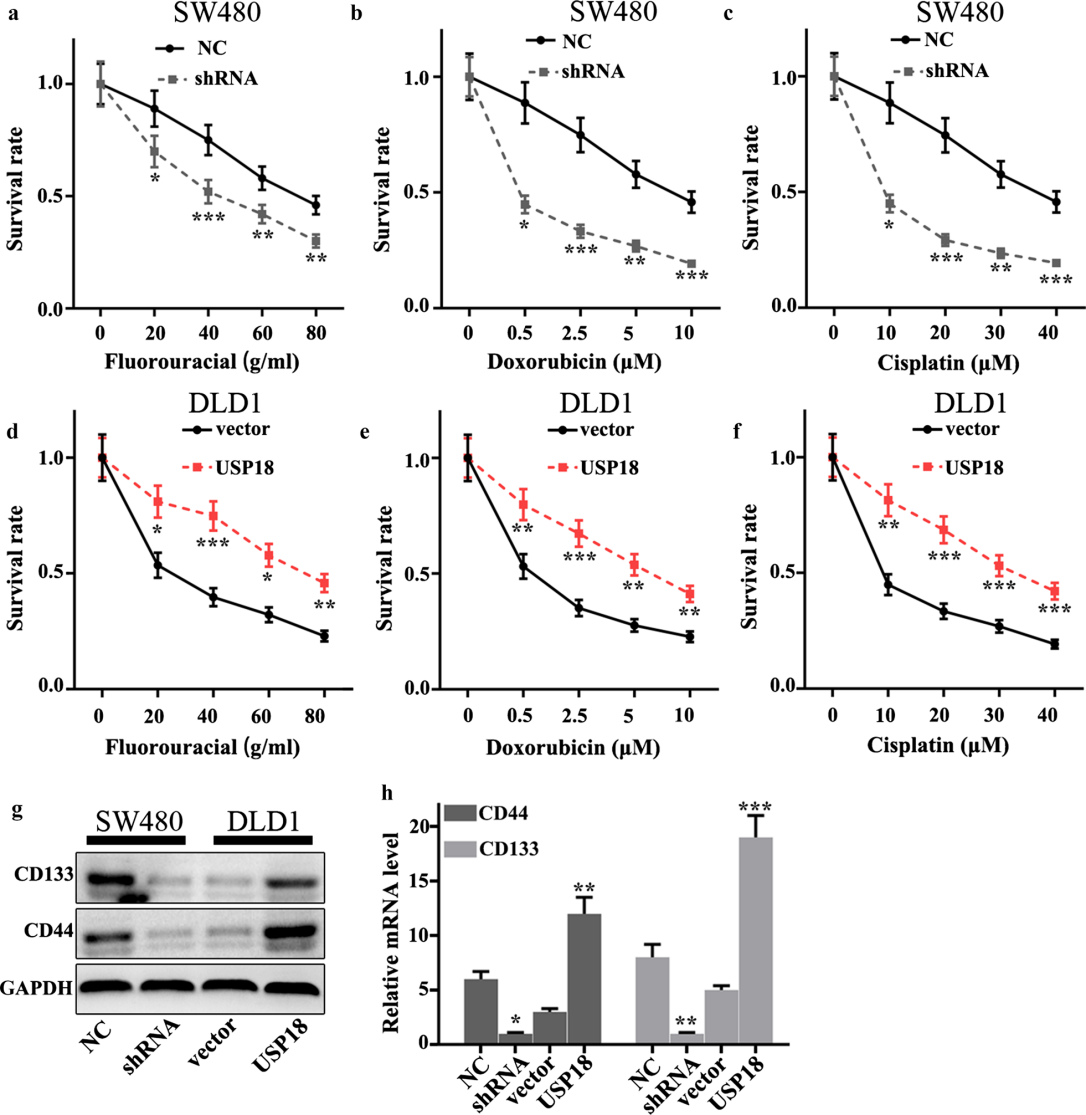
Effect of USP18 on chemotherapy sensitivity of colorectal cancer cells. **a**–**f** Cellular chemotherapy sensitivity experiment on three common chemotherapeutics (fluorouracial, doxorubicin and cisplatin) in USP18 knocked-down SW480 cells and USP18 over-expressed DLD1 cells. **g**–**h** Western blot and qRT-PCR analysis of stem cell-related proteins (CD133/CD44). *P < 0.05, **P < 0.01, ***P < 0.001

We investigated whether the changes of USP18 expression could affect the chemotherapy sensitivity of colorectal cancer cells using three common chemotherapeutic molecular drugs, including fluorouracial, doxorubicin and cisplatin Different concentrations of fluorouracial (0, 20, 40, 60, and 80 g/mL), doxorubicin (0, 0.5, 2.5, 5, and 10 µM), and cisplatin (0, 10, 20, 30, and 40 µM) were applied in this study. The results suggested that USP18 knockdown in SW480 cells could significantly decrease cell survival in three different drug treatments compared to normal SW480 cells (P < 0.05) (Fig. 4a–c). However, USP18 overexpression in DLD1 cells could effectively promote cell survival in three different drug treatments compared to normal DLD1 cells (P < 0.05) (Fig. 4d–f). These results revealed that USP18 knockdown in SW480 cells and USP18 overexpression in DLD1 cells were closely related to cell survival rate changes caused by drug treatments. Moreover, we evaluated the protein and mRNA expression of stem cell-related biomarkers (i.e., CD44 and CD133). It is indicated that USP18 knockdown in SW480 cells could significantly decrease protein and mRNA expression of stem cell-related biomarkers, while USP18 overexpression in DLD1 cells could effectively promote protein and mRNA expression of stem cell-related biomarkers (P < 0.001) (Fig. 4g, h). The above results suggested that USP18 expression was closely related to stem cell ability.

### USP18 regulated Snail1 expression through ubiquitination

**Fig. 5:**
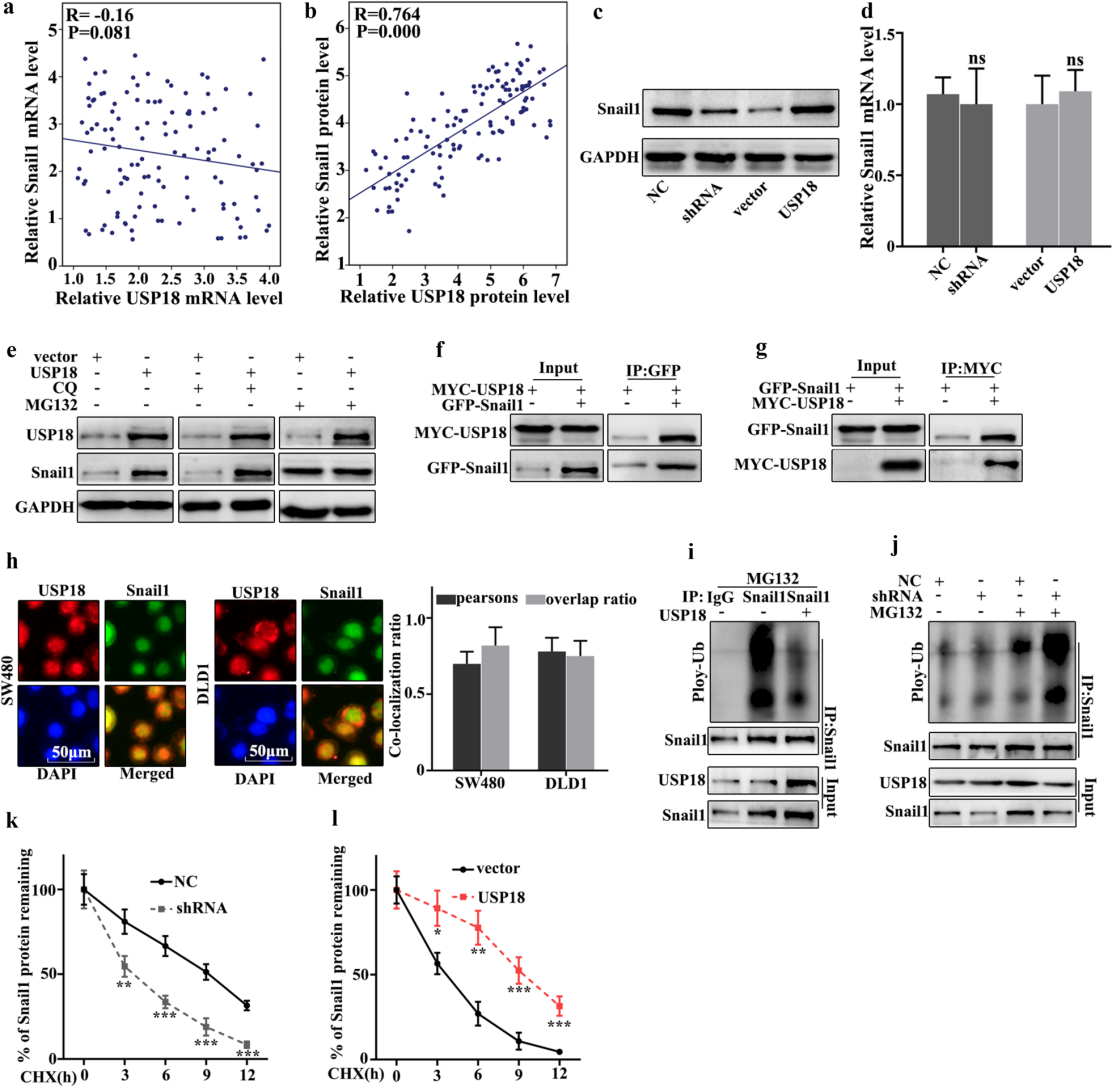
USP18 regulated Snail1 expression through ubiquitination. **a** Correlation verification between USP18 and Snail1 mRNA in 60 colon cancer specimens. **b** The correlation between USP18 and Snail1 protein expression was calculated in 60 pairs of colon cancer specimens. **c**–**d** Western blot and qRT-PCR analysis the relationships between USP18 and Snail1. **e** Snail1 protein expression analysis with CQ and MG132 treatments. **f** Co-precipitation analysis of USP18 and Snail1 protein in SW480 cell. **g** Co-localization of immunofluorescence analysis of USP18 and Snail1 in SW480 and DLD1 cell. **h** Co-precipitation of USP18 and Snail1 in the 293T cell. **i** Ubiquitination testing of USP18 in DLD1 cell line. **j** Ubiquitination testing of USP18 in SW480 cell line. **k**, **l** Cycloheximide (protein degradation rate) analysis showed that USP18 could inhibit Snail1 protein degradation. *P < 0.05, **P < 0.01, ***P < 0.001

In this study, we further studied the potential relationships between the expressions of USP18 and Snail1. We further analyzed the potential relationships of the mRNA expressions of USP18 and Snail1 in 60 clinical samples (Fig. 5a, b). The results suggested that no correlation could be observed between the mRNA expressions of USP18 and Snail1 in clinical samples. However, a significant correlation could be observed between the protein expressions of USP18 and Snail1 (P = 0.000). Moreover, western blot and qRT-PCR were employed to investigate the protein and mRNA expressions of Snail1 in USP18 knocked-down SW480 cells, and USP18 over-expressed DLD1 cells. Figure 5c showed that Snail1 protein expression was lower in USP18 knocked-down SW480 cells than in normal SW480 cells, but Snail1 protein expression was more abundant in USP18 over-expressed DLD1 cells than in normal DLD1. It was notable that no difference of Snail1 mRNA abundance could be detected in USP18 knocked-down SW480 cells and USP18 over-expressed DLD1 cells (Fig. 5d). The above results suggested that USP18 could affect the Snail1 protein expression but not Snail1 mRNA level. Moreover, we investigated the potential relationships between USP18 ubiquitination and Sanil1 in USP18 over-expressed DLD1 cells. MG132 is the inhibitor of proteasome degradation pathway in the cell and Chloroquine (CQ) is an inhibitor of autophagolysosomal degradation pathway. Therefore, we employed MG132 and CQ to study the protein expressions of USP18 and Snail1 in USP18 over-expressed DLD1 cells (Fig. 5e). The results suggested that MG132 could effectively increase Snail1 protein expression but CQ exerted no effect. Therefore, we speculated that USP18 could regulate Snail1 expression through the ubiquitination but not the cellular autophagy pathway. Moreover, we examined the potential interaction between USP18 protein and Snail1 protein in cellular using Co-immunoprecipitation (Co-IP). Figure 5f showed that USP18 protein could interact with Snail protein. Moreover, we carried out the forward and reverse protein Co-IP of USP18 protein and Snail1 protein in USP18 over-expressed DLD1 cells. Figure 5g further identified the interaction between USP18 protein and Snail1 protein. Furthermore, immunofluorescence colocalization analysis of USP18 protein and Snail1 revealed that the spatial distribution of both molecular was overlapping. The above result further illustrated the mutual combination between USP18 protein and Snail1 in USP18 over-expressed DLD1 cells (Fig. 5h). Furthermore, we employed ubiquitin detection in USP18 knocked-down SW480 cells and USP18 over-expressed DLD1 cells (Fig. 5i, j). The results suggested that USP18 knocked-down SW480 cells could significantly promote the degradation of the remaining Snail1 protein in cells compared to that in normal SW480 cells (P < 0.01). Meanwhile, USP18 over-expressed DLD1 cells could significantly decrease the degradation of the remaining Snail1 protein in cells compared to that in normal DLD1 cells (P < 0.01). Furthermore, we have further employed cycloheximide (CHX) to study the relationships between protein expressions of USP18 and Snail in USP18 knocked-down SW480 cells and USP18 over-expressed DLD1 cells. Figure 5k, l suggested that USP18 over-expressed DLD1 cells could significantly promote the remaining Snail1 protein in cells compared to normal DLD1 cells (P < 0.01). Meanwhile, USP18 knocked-down SW480 cells could significantly decrease the remaining Snail1 protein in cells compared to normal SW480 cells (P < 0.01).

### Snail1 could effectively reverse the influence of USP18 on cell proliferation, migration, invasion, and EMT of CRC cells

**Fig. 6:**
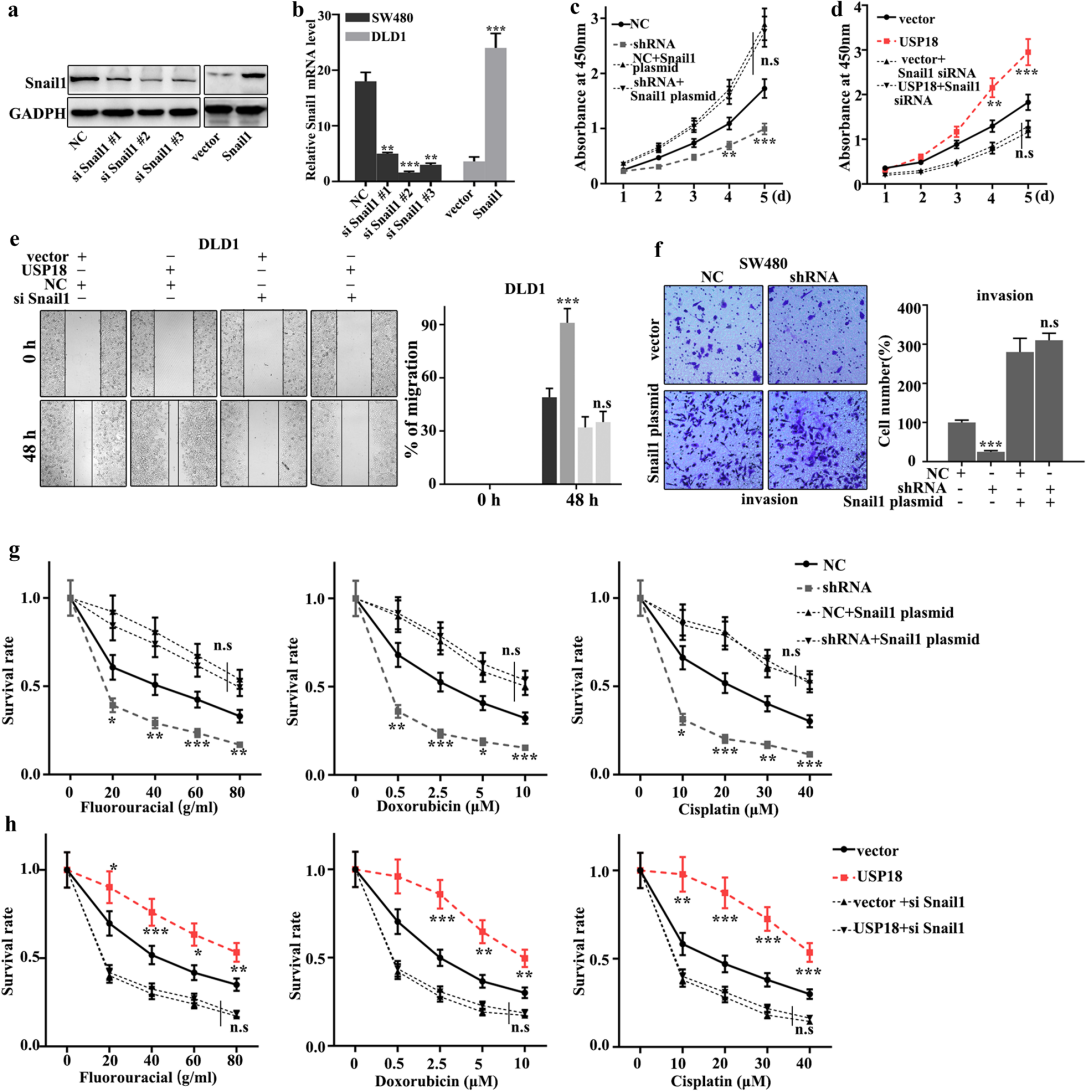
Snail1 could effectively reverse the influence of USP18 on cell proliferation, migration, invasion, and EMT of CRC cells. **a**, **b** Western blot and qRT-PCR analysis of the optimal interference sequence and overexpression sequence of Snail 1 siRNA. **c**, **d** CCK8 analysis of the USP18 knocked-down SW480 cells and USP18 over-expressed DLD1 cells with Snail1 siRNA and overexpression plasmid treatments. **e**, **f** Scratch experiment analysis of the USP18 over-expressed DLD1 cells and USP18 knocked-down SW480 cells with Snail1 siRNA and overexpression plasmid treatments. **g**, **h** Three common chemotherapeutics (fluorouracial, doxorubicin and cisplatin) for cell survival rate analysis of the USP18 knocked-down SW480 cells and USP18 over-expressed DLD1 cells with Snail1 siRNA and overexpression plasmids treatments. *P < 0.05, **P < 0.01, ***P < 0.001

In this study, we further performed the rescue experiment to demonstrate the relationship between USP18 and Snail1. We introduced Snail1 knockdown and overexpression treatment in USP18 knockdown-treated SW480 cells and USP18 overexpression-treated DLD1 cells. Figure 6a, b showed that siSnail1 #3 and Snail1 vector were effective to establish knockdown and overexpression models of Snail1. Meanwhile, Fig. 6c, d suggested that knockdown and overexpression treatment of Snail1 gene treatments could effectively inhibit and promote cell proliferation caused by USP18 overexpression and knockdown treatments, respectively. Moreover, we studied the cell scratch of the Snail1 knockdown and overexpression treatments in DLD1 cells, which had been treated with USP18 overexpression and USP18 knockdown, respectively. Figure 6e showed that the Snail1 knockdown could effectively reverse the changes in cell scratch caused by USP18 knockdown (P < 0.01). Meanwhile, Snail1 overexpression could effectively reverse the changes of invasion caused by USP18 overexpression in SW480 cells (P < 0.01) (Fig. 6f). Furthermore, we studied the effect of USP18 and Snail1 on chemotherapy sensitivity of colorectal cancer. Figure 6g, h suggested that Snail1 overexpression and knockdown treatments could effectively reverse the changes in cell survival rate caused by USP18 knockdown and overexpression treatment in the chemotherapy sensitivity analysis (P < 0.01). In summary, knockdown and overexpression of Snail1 could effectively reverse the influence of USP18 on cell proliferation, migration, invasion, and EMT.

## Discussion

In China, colorectal cancer remains as one of the most common malignant tumors in the digestive system, with a third incidence rate among all tumors and being increasing over the past decades due to environmental and dietary factors [22, 23]. Studies have found the relationship between its incidence and intestinal polyposis, chronic stress and inflammation, and a family history of cancer [24]. Previous studies suggested that the main cause of death was the invasion and migration of advanced colorectal cancer [25]. Improving diagnostic techniques, including surgery, chemotherapy and radiotherapy treatments, could help detect tumors early and improve patient survival. Better understanding the pathogenesis progression may provide new therapeutic strategies for the prevention and treatment of CRC.

USP18 is an effective regulator of epidermal growth factor receptors (EGFR) [19], and the low expression of it could lead to a down-regulated expression of carcinogenic targets thus decreasing cell proliferation and increasing cell apoptosis [26]. In MCF-7 cells and glioblastoma, knocking out USP18 can induce apoptosis of tumor cells [27, 28]. The low expression of USP18 can significantly reduce the metastasis and invasion of lung cancer cells [29]. However, what role USP18 plays in the progression of CRC has not been reported.

Ubiquitination plays an important role in several biological processes including metabolism, protein degradation, cellular localization, inflammatory immunity, transcription regulation, and cell cycle [30]. Meanwhile, ubiquitination is closely linked with the regulation of tumors. It was reported that over-expressed DUSP4 could promote chemotherapy-induced apoptosis. In addition, silence of DUSP4 could activate the Ras-ERK signaling pathway and further promote the proliferation and migration of tumor cells [31]. However, the role of USP18 in the regulation of tumor cells is poorly understanded. In this study, we demonstrated that the change of USP18 expression was closely related to the invasion, migration, and proliferation of CRC cells. Therefore, USP18 may provide a potential target for the treatment of CRC. Recent reports indicated that abnormal expression of USP18 in CRC tissues was associated with a poorer prognosis [17]. Those results are consistent with our findings, which showed that the expression of USP18 was higher in CRC tissues than in adjacent tissues, and overexpression or knockout of USP18 could affect the proliferation, and migration of CRC cells. Therefore, USP18 might be a biomarker for the diagnosis of CRC.

Snail1 is a nuclear transcription factor that can control the transcription efficiency of DNA to messenger RNA [32]. Previous study suggested that Snail1 can inhibit the expression of the downstream gene Cyclin D2 and promote cell survival [33]. Meanwhile, it was suggested that Snail1 can upregulate the expression of myosin that could promote the migration of tumor cells [34]. However, whether USP18 could affect the proliferation, migration and invasion of CRC cells through targeting Snail1 remains unclear.

EMT process has been believed to act a key role affecting tumor metastasis. EMT is characterized by decreased expression of epithelial proteins such as E-Cadherin, and increased level of mesenchymal protein such as vimentin. Low E-Cadherin expression means decreased of epithelial connexin, and further facilitate tumor metastasis. In this study, we found that E-Cadherin, Neadherin, and Vimentin were remarkably influenced in the overexpression and knockdown models of USP18. Protein stability is mainly affected by proteasome degradation pathways and autophagolysosomal degradation pathways.

Snail1 is an important transcription factor of EMT, and the expression level of it is linked with the invasion, migration, and apoptosis of tumor cells. Snail1 is believed to be an important factor affecting the neural tube and development of mesoderm, but also plays an important role in tumor metastasis. Snail1 is the most important E-cadherin transcriptional repressor, and it could down-regulate the expression of claudins and occludins protein. Our results revealed that DUSP18 and Snail1 could regulate EMT of CRC through E-caderin, N-caderin, and Vitmentin. Snail1 can directly interact with USP18 in cellular.

In this study, it was notable that Snail1 expression was significantly affected in USP18 over-expressed or knocked-down cells. Snail1 could directly interact with USP18 in cells. Moreover, USP18 could reduce Snail1 protein expression without affecting its transcription. Moreover, our results suggested that USP18 affected the protein degradation pathway of Snail1 through ubiquitination modification. Meanwhile, knockdown and overexpression of Snail1 could affect cell migration and invasion, and EMT-related molecules including E-cadherin, N-cadherin, and Vimentin. We proved that Snail1 could effectively reverse the influence of USP18 on cell proliferation, migration, invasion, and EMT of CRC cells.

## Conclusions

We demonstrated that high USP18 expression could be detected in colorectal cancer tissues and cells. USP18 might regulate the cell proliferation, invasion, migration, and EMT process of CRC cells through targeting Snail1 ubiquitylation degradation pathway. This might provide a novel thought for the prevention and treatment of CRC by targeting USP18/Snail1.

## Supplementary information

**Additional file 1.** Sequence of primers for Quantitative reverse transcription-PCR.

## Data Availability

All data generated or analysed during this study are included in this published article.

## Abbreviations

CRC: Colorectal cancer
USP18: Ubiquitin-specific peptidase 18
qRT-PCR: Quantitative real-time PCR test
EMT: Epithelial-mesenchymal transformation
CQ: Chloroquine
Co-IP: Co-immunoprecipitation
CHX: Cycloheximide
EGFR: Epidermal growth factor receptors
