# Supplementary material for: USP18 directly regulates Snail1 protein through ubiquitination pathway in colorectal cancer
Source: Cancer Cell Int. 2020 Jul 28;20:346. doi: 10.1186/s12935-020-01442-1 (PMC7389447; doi:10.1186/s12935-020-01442-1)
Supplement: Supplementary file 1 — Additional file 1. Sequence of primers for Quantitative reverse transcription-PCR. [file 12935_2020_1442_MOESM1_ESM.doc]

| **Additional file 1: Table S1.** Sequence of primers for Quantitative reverse transcription -PCR | | |
| --- | --- | --- |
| Gene | Forward primer (5’------3’) | Reverse primer(5’------3’) |
| USP18 | AAGGTCTTCGGACGGCCTA | GCAGCGAAAACTTGAGGGTAT |
| E-cadherin | CGAGAGCTACACGTTCACGG | GGGTGTCGAGGGAAAAATAGG |
| Vimentin | CGAAACTTCTCAGCATCACG | GCAGAAAGGCACTTGAAAGC |
| N-cadherin | TCAGGCGTCTGTAGAGGCTT | ATGCACATCCTTCGATAAGACTG |
| CD133 | AGTCGGAAACTGGCAGATAGC | GGTAGTGTTGTACTGGGCCAAT |
| CD44 | CTGCCGCTTTGCAGGTGTA | CATTGTGGGCAAGGTGCTATT |
| Snail1 | TCGGAAGCCTAACTACAGCGA | AGATGAGCATTGGCAGCGAG |
| GAPDH | AGAAGGCTGGGGCTCATTTG | AGGGGCCATCCACAGTCTTC |
